# Enhanced glycolysis in granulosa cells promotes the activation of primordial follicles through mTOR signaling

**DOI:** 10.1038/s41419-022-04541-1

**Published:** 2022-01-27

**Authors:** Xiaodan Zhang, Wenbo Zhang, Zhijuan Wang, Nana Zheng, Feifei Yuan, Biao Li, Xuelan Li, Ling Deng, Min Lin, Xin Chen, Meijia Zhang

**Affiliations:** 1 State Key Laboratory for Agrobiotechnology, College of Biological Sciences, China Agricultural University, 100193 Beijing, China; 2 Division of Cell, Developmental and Integrative Biology, School of Medicine, South China University of Technology, 510006 Guangzhou, China; 3 The Center for Reproductive Medicine, Shunde Hospital, Southern Medical University (The First People’s Hospital of Shunde), 528300 Foshan, Guangdong China

**Keywords:** Physiology, Endocrine reproductive disorders

## Abstract

In mammals, nonrenewable primordial follicles are activated in an orderly manner to maintain the longevity of reproductive life. Mammalian target of rapamycin (mTOR)-KIT ligand (KITL) signaling in pre-granulosa cells and phosphatidylinositol 3-kinase (PI3K)-protein kinase B (Akt)-forkhead Box O3a (FOXO3a) signaling in oocytes are important for primordial follicle activation. The activation process is accompanied by the enhancement of energy metabolism, but the causal relationship is unclear. In the present study, the levels of glycolysis-related proteins GLUT4, HK1, PFKL, and PKM2 were significantly increased in granulosa cells but were decreased in oocytes during the mouse primordial-to-primary follicle transition. Both short-term pyruvate deprivation in vitro and acute fasting in vivo increased the glycolysis-related gene and protein levels, decreased AMPK activity, and increased mTOR activity in mouse ovaries. The downstream pathways Akt and FOXO3a were phosphorylated, resulting in mouse primordial follicle activation. The blockade of glycolysis by 2-deoxyglucose (2-DG), but not the blockade of the communication network between pre-granulosa cells and oocyte by KIT inhibitor ISCK03, decreased short-term pyruvate deprivation-promoted mTOR activity. Glycolysis was also increased in human granulosa cells during the primordial-to-primary follicle transition, and short-term pyruvate deprivation promoted the activation of human primordial follicles by increasing the glycolysis-related protein levels and mTOR activity in ovarian tissues. Taken together, the enhanced glycolysis in granulosa cells promotes the activation of primordial follicles through mTOR signaling. These findings provide new insight into the relationship between glycolytic disorders and POI/PCOS.

## Introduction

In mammals, the nonrenewable primordial follicle pool is established around the time of birth [1]. In each wave, a limited number of primordial follicles are activated into the growing stage, while the remaining follicles are maintained in the quiescent state [2]. Orderly activation of primordial follicles is critical to maintain the longevity of reproductive life [3]. Primary ovarian insufficiency (POI) and polycystic ovary syndrome (PCOS) are the most prevalent ovarian diseases affecting women’s fertility and health. In POI patients, the altered recruitment of primordial follicles causes a decrease in ovarian reserve [4]. In PCOS patients, the accumulation of preantral small follicles may be caused by an increase in primordial follicle activation and the inability of antral follicle growth [5]. Therefore, elucidation of the molecular mechanism of primordial follicle activation is crucial for the diagnosis and treatment of ovarian diseases.

The primordial follicle consists of a dormant oocyte and a single layer of squamous pre-granulosa cells. Upon follicle activation, the size of the oocytes increases, and the morphology of the pre-granulosa cells become cuboidal [6]. The mammalian target of rapamycin (mTOR) is one of the major signaling pathways. The activation of mTOR signaling in pre-granulosa cells can increase KIT ligand (KITL) secretion. KITL binds to KIT on the surface of the oocyte to activate phosphatidylinositol 3-kinase (PI3K)-protein kinase B (Akt) signaling in oocytes, and then forkhead Box O3a (FOXO3a) is phosphorylated and transported out of the nucleus [7–9]. The inhibition of oocyte growth is lifted, and the follicle begins to grow. Other molecules have also been reported to be involved in the activation of primordial follicles by regulating the above signaling pathways, including mitogen-activated protein kinase (MAPK3/1), E-cadherin, and histone deacetylase 6 (HDAC6) [10–12].

It has been reported that energy metabolism is increased during the primordial-to-primary follicle transition [13, 14]. In addition, glycolysis is increased as follicles progress from secondary to preovulatory stages [15–17]. Thus, follicular development is accompanied by an increase in glycolysis, which provides the main energy source. The main sensor of cellular energy status is AMP-activated protein kinase (AMPK), which is highly conserved in all eukaryotic species [18]. In general, AMPK maintains the energy balance by regulating the anabolic and catabolic processes [19]. Acute AMPK activation in response to energy deficiency favors glucose uptake and glycolysis to promote ATP restoration [20]. When the energy is sufficient, mTOR signaling is activated by a decrease in AMPK activity and then it performs a variety of physiological functions [21].

The primordial follicle activation is accompanied by an enhancement of energy metabolism. However, the relationship between glycolysis (the main energy source of the follicles) and primordial follicle activation is unclear. In this study, short-term pyruvate deprivation and/or acute fasting increased glycolysis in the granulosa cells and then activated mTOR signaling, resulting in mouse and human primordial follicle activation.

## Results

### The expression pattern of glycolysis-related genes and proteins in neonatal mouse ovaries

**Fig. 1:**
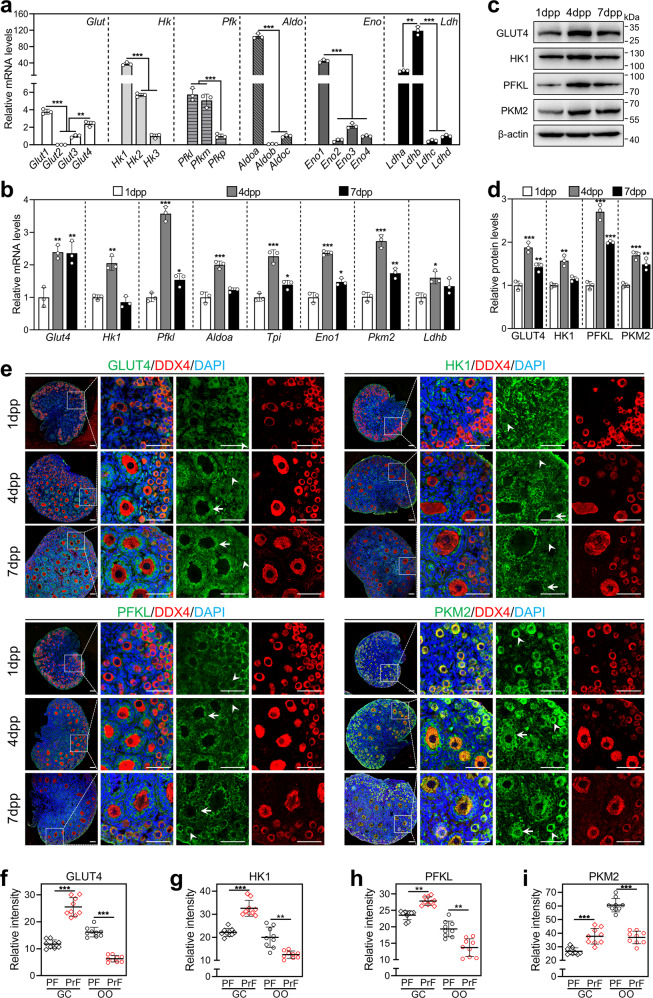
The expression pattern of glycolysis-related genes and proteins in neonatal mouse ovaries. **a** The mRNA levels of the isoforms in each family of *Glut*, *Hk*, *Pfk*, *Aldo*, *Eno*, and *Ldh* in mouse ovaries at 4 dpp. The mRNA values of *Glut3*, *Hk3*, *Pfkp*, *Aldoc, Eno4,* and *Ldhd* were set as 1. (*n* = 3 independent experiments). Bars indicate the mean ± SD. **P < 0.01 and ***P < 0.001. **b** The mRNA levels of *Glut4*, *Hk1*, *Pfkl*, *Aldoa*, *Eno1*, *Tpi*, *Pkm2*, and *Ldhb* in the ovaries at 1, 4 and 7 dpp. (*n* = 3 independent experiments). Bars indicate the mean ± SD. *P < 0.05, **P < 0.01, and ***P < 0.001 vs. 1 dpp group. **c**, **d** The protein levels of GLUT4, HK1, PFKL, and PKM2 in the ovaries at 1, 4, and 7 dpp. (*n* = 3 independent experiments). β-actin was used as an internal control. Bars indicate the mean ± SD. **P < 0.01 and ***P < 0.001 vs. 1 dpp group. **e** Immunofluorescence stain of GLUT4, HK1, PFKL, and PKM2 (green) in the ovaries at 1, 4, and 7 dpp. (*n* = 3 independent experiments). The cytoplasm of oocytes was stained with the oocyte-specific marker DDX4 (red), and the nuclei were counterstained by DAPI (blue). The arrowheads and the arrows show the primordial and primary follicles, respectively. Scale bars: 50 μm. **f**–**i** The intensity of GLUT4 (**f**), HK1 (**g**), PFKL (**h**), and PKM2 (**i**) fluorescent signals in granulosa cells (GC) and oocytes (OO) of primordial follicles (PF) and primary follicles (PrF). (*n* = 9 sections from 7 dpp ovaries. The total number of 45 follicles was scored in each group). Bars indicate the mean ± SD. **P < 0.01 and ***P < 0.001. The representative images are shown.

We first compared the mRNA levels of the isoforms in each family of *Glut* (glucose transporter), *Hk* (hexokinase), *Pfk* (phosphofructokinase), *Aldo* (aldolase), *Eno* (enolase), and *Ldh* (lactate dehydrogenase) in mouse ovaries at 4 dpp, in which the primordial follicle pool was established and the first wave of primordial follicles was activated [2]. The results showed that *Glut1/Glut4*, *Hk1*, *Pfkl*, *Aldoa*, *Eno1*, and *Ldhb* were highly expressed (Fig. 1a). During ovarian development from 1 dpp to 4 dpp, the mRNA levels of *Glut4*, *Hk1*, *Pfkl*, *Aldoa*, *Eno1*, *Tpi* (triosephosphate isomerase, the sole isoform in its family), *Pkm2* (pyruvate kinase M2, the sole isoform in its family) and *Ldhb* were significantly increased (Fig. 1b). The protein levels of GLUT4 and glycolysis rate-limiting enzymes (HK1, PFKL, and PKM2) were also significantly increased in the ovaries at 4 dpp compared with those in the ovaries at 1 dpp (Fig. 1c, d). The increase in these glycolysis-related genes and proteins indicates that glycolysis is enhanced [15, 17], which is closely related to the activation of primordial follicles.

Next, we detected the cellular localization and expression dynamics of these glycolysis-related proteins in neonatal mouse ovaries. In primordial follicles, GLUT4, HK1, PFKL, and PKM2 were localized in the cytoplasm of pre-granulosa cells and oocytes (Fig. 1e). In growing follicles, these proteins were localized in the cytoplasm of granulosa cells, and only PKM2 was also localized in the cytoplasm of oocytes (Fig. 1e). The intensity of the fluorescent signals was analyzed in the sections from 7 dpp ovaries, which contained both primordial and primary follicles. All of these proteins were significantly increased in granulosa cells but were decreased in oocytes during the primordial-to-primary follicle transition (Fig. 1f–i). These results suggest that glycolysis is increased in granulosa cells but is decreased in oocytes during mouse primordial follicle activation. Even though *Glut1* mRNA levels were the highest in mouse ovaries at 4 dpp, its protein was predominantly localized in stromal cells (including theca cells) rather than in preantral follicle cells (Supplementary Fig. S1). In adult ovaries, GLUT1 was also strongly expressed on the plasma membrane of antral follicle cells (Supplementary Fig. S2).

### Short-term pyruvate deprivation promotes the activation of mouse primordial follicles in vitro

**Fig. 2:**
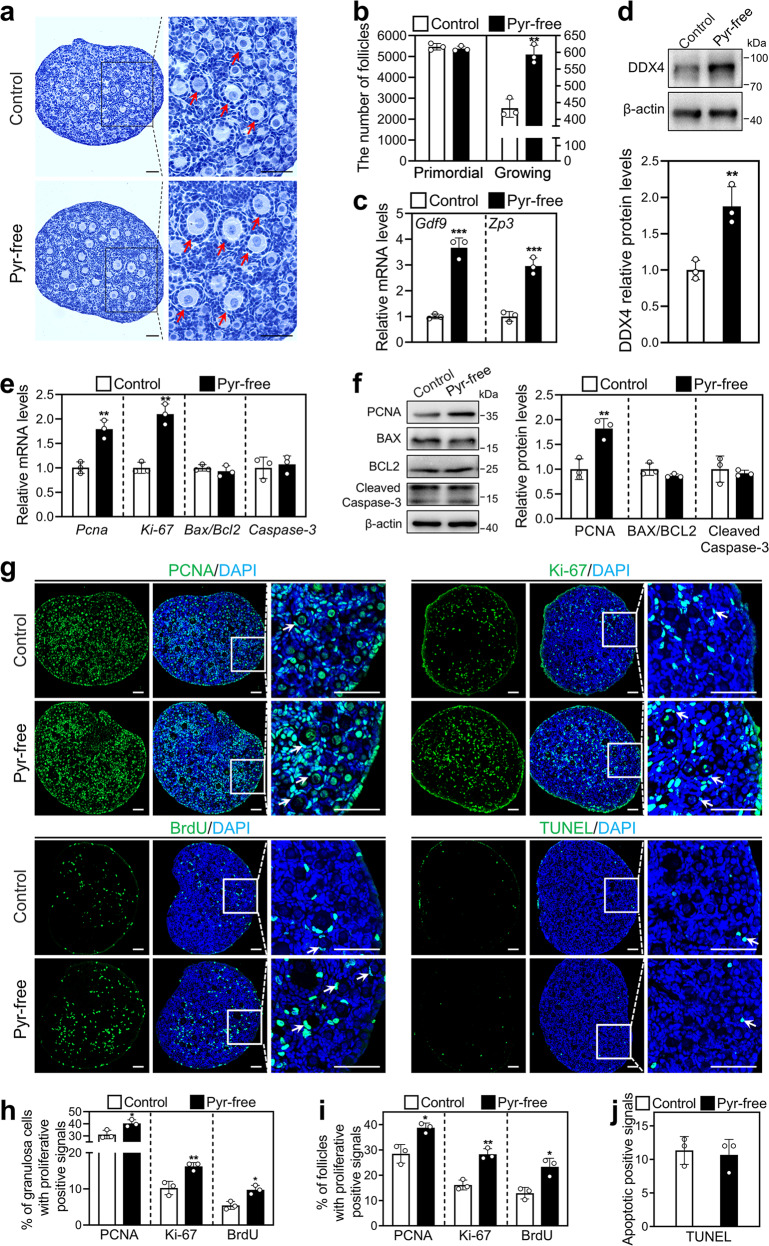
Effect of short-term pyruvate deprivation on mouse primordial follicle activation in vitro. Ovaries at 2 dpp were cultured in standard (control) or pyruvate-free (pyr-free) medium for 1 day (**e**–**j**) or 2 days (**a**–**d**). **a**, **b** Morphological comparison of the ovaries (**a**) and the number of primordial and growing follicles (arrows, **b**) in the control and pyruvate-free group. Nuclei were stained by hematoxylin. Scale bars: 50 μm. **c** The mRNA levels of *Gdf9* and *Zp3* in the control and pyruvate-free group. **d** DDX4 protein levels in the control and pyruvate-free group. **e** The mRNA levels of *Pcna*, *Ki-67*, *Bax*/*Bcl-2*, and *Caspase-3* in the control and pyruvate-free group. **f** The protein levels of PCNA, BAX, BCL-2, and Cleaved Caspase-3 in the control and pyruvate-free group. **g** Immunofluorescence stain of PCNA, Ki-67, BrdU, and TUNEL (green) in the control and pyruvate-free group. DAPI, blue. Scale bars: 50 μm. **h**–**j** The percentage of granulosa cells (**h**) and primordial follicles (**i**) with PCNA-, Ki-67-, or BrdU-positive signals, and the number of cells with TUNEL-positive signals (**j**) in the control and pyruvate-free group. All the experiments were repeated three times, and the representative images are shown. In western blot results, β-actin was used as an internal control. Bars indicate the mean ± SD. *P < 0.05, **P < 0.01, and ***P < 0.001 vs. control.

Pyruvate, the end product of glycolysis, is the main energy substrate of mitochondrial oxidation. We studied the effect of pyruvate deprivation on mouse primordial follicle activation. Interestingly, pyruvate-free treatment (593.33) significantly increased the number of growing follicles in contrast to the control (433.33; Fig. 2a, b), and slightly decreased the number of primordial follicles compared with that in the control group. Pyruvate-free treatment also significantly increased the mRNA levels of *Gdf9* and *Zp3* (oocyte developmental markers) and the protein levels of DDX4 (a cytosolic marker of oocytes; Fig. 2c, d). These results demonstrated that short-term pyruvate deprivation promotes mouse primordial follicle activation in vitro.

### Short-term pyruvate deprivation increases the proliferation of mouse granulosa cells

The onset of pre-granulosa cell proliferation is a characteristic of primordial follicles entering the growth states [6]. The neonatal mouse ovaries were cultured in standard or pyruvate-free medium for 1 day and detected for cell proliferation and apoptosis. Pyruvate-free treatment significantly increased the mRNA levels of *Pcna* and *Ki-67*, as well as the protein levels of PCNA (Fig. 2e, f). The percentage of granulosa cells with PCNA-, Ki-67- and BrdU-positive signals in the pyruvate-free group was significantly increased compared with that in the control group (PCNA: 40.38% vs. 31.13%; Ki-67: 16.23% vs. 10.24%; BrdU: 9.76% vs. 5.42%; Fig. 2g, h). And, the number of primordial follicles with proliferation-positive signals in the pyruvate-free group was also significantly increased compared with that in the control group (PCNA: 38.76% vs. 28.47%; Ki-67: 28.31% vs. 16.15%; BrdU: 23.33% vs. 12.90%; Fig. 2g, i). However, the mRNA (*Bax*/*Bcl-2* and *Caspase-3*) and protein (BAX/BCL-2 and Cleaved Caspase-3) levels were not different between the pyruvate-free group and the control (Fig. 2e, f). Furthermore, pyruvate-free treatment had no influence on the number of cells with TUNEL-positive signals (Fig. 2g, j). These results indicate that short-term pyruvate deprivation promotes pre-granulosa cell proliferation but has no effect on cell apoptosis.

### Short-term pyruvate deprivation promotes glycolysis and mTOR signaling in cultured mouse ovaries

**Fig. 3:**
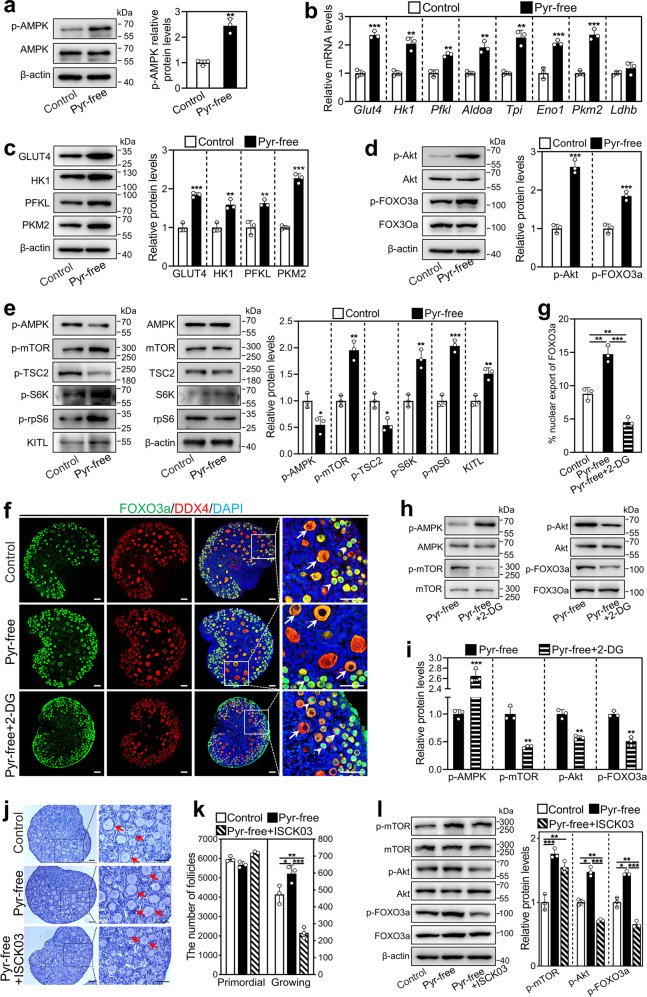
Effect of short-term pyruvate deprivation on glycolysis and mTOR signaling in cultured mouse ovaries. Ovaries at 2 dpp were cultured in standard (control) or pyruvate-free (pyr-free) medium for 6 h (**a**), 12 h (**b**), 24 h (**c**, **e**) and 48 h (**d**, **f**, **g**), or cultured in pyruvate-free medium supplemented with 10 mM 2-DG (pyr-free + 2-DG) or 5 μM ISCK03 (pyr-free + ISCK03) for 48 h (**f**–**l**). **a** The protein levels of p-AMPK in the control and pyruvate-free group. **b** The mRNA levels of *Glut4*, *Hk1*, *Pfkl*, *Aldoa*, *Eno1*, *Tpi*, *Pkm2*, and *Ldhb* in the control and pyruvate-free group. **c** The protein levels of GLUT4, HK1, PFKL, and PKM2 in the control and pyruvate-free group. **d** The protein levels of p-Akt and p-FOXO3a in the control and pyruvate-free group. **e** The protein levels of p-AMPK, p-mTOR, p-TSC2, p-S6K, p-rpS6, and KITL in the control and pyruvate-free group. **f**, **g** The localization of FOXO3a in oocyte nuclear (arrowheads) and cytoplasm (arrows, **f**) and the percentage of oocytes with FOXO3a nuclear export (**g**) in each section in the control, pyruvate-free and pyruvate-free + 2-DG groups. FOXO3a, green; DDX4, red; DAPI, blue. Scale bars: 50 μm. **h**, **i** The protein levels of p-AMPK, p-mTOR, p-Akt, and p-FOXO3a in the pyruvate-free (as control) and pyruvate-free + 2-DG group. 2-DG, 2-deoxyglucose. **j**, **k** Morphological comparison of the ovaries (**j**) and the number of primordial and growing follicles (arrows. **k**) in the control, pyruvate-free and pyruvate-free + ISCK03 groups. Nuclei were stained by hematoxylin. Scale bars: 50 μm. **l** The protein levels of p-mTOR, p-Akt and p-FOXO3a in the control, pyruvate-free and pyruvate-free + ISCK03 groups. All the experiments were repeated three times, and the representative images are shown. In western blot results, total AMPK, mTOR, TSC2, S6K, rpS6, Akt, and FOXO3a were used as the corresponding internal control for p-AMPK, p-mTOR, p-TSC2, p-S6K, and p-rpS6, p-Akt, and p-FOXO3a, respectively, and β-actin was used as the internal control for GLUT4, HK1, PFKL, PKM2, and KITL. Bars indicate the mean ± SD. *P < 0.05, **P < 0.01, and ***P < 0.001.

Cells grown in the pyruvate-free medium will rapidly secrete pyruvate into the culture medium [22], resulting in a temporary energy shortage inside the cells. We cultured mouse ovaries (2 dpp) in a standard or pyruvate-free medium and detected the activity of the energy sensor AMPK [18]. The levels of phosphorylated AMPK (p-AMPK) were significantly increased after 6 h of culture in the pyruvate-free group compared with the control group (Fig. 3a). AMPK activation will result in an increase in glucose uptake and subsequent glycolysis to promote ATP restoration [20]. We therefore tested the glycolysis-related gene and protein levels in the ovaries after 12 and 24 h of culture, respectively. The levels of mRNAs (*Glut4*, *Hk1*, *Pfkl*, *Aldoa*, *Eno1*, *Tpi*, and *Pkm2*) and proteins (GLUT4, HK1, PFKL, and PKM2) were significantly increased in the pyruvate-free group compared with the control (Fig. 3b, c). These results demonstrate that short-term pyruvate deprivation increases glycolysis by activating AMPK in mouse ovaries.

In general, an increase in glycolysis by the acute activation of AMPK in turn reduces AMPK activity to normal [19]. Interestingly, the levels of p-AMPK in the pyruvate-free group were significantly lower than the control after 24 h of culture (Fig. 3e), possibly because of the continued increase in glycolysis in the pyruvate-free group further decreased AMPK activity (Supplementary Fig. S3). A decrease in AMPK activity will activate mTOR pathways [21]. Indeed, the protein levels of p-mTOR, p-S6K, and p-rpS6 were significantly increased, and p-TSC2 was significantly decreased in the pyruvate-free group compared with the control after 24 h of culture (Fig. 3e). In contrast, the blockade of glycolysis by 2-deoxyglucose (2-DG, an inhibitor of glycolysis) in the pyruvate-free + 2-DG group significantly increased the p-AMPK levels and decreased the p-mTOR levels compared with those in the pyruvate-free group after 48 h of culture (Fig. 3h, i). Thus, the enhanced glycolysis induced by short-term pyruvate deprivation activates mTOR signaling by decreasing AMPK activity in mouse ovaries.

Next, we detected the levels of mTOR downstream signaling molecules that are required for primordial follicle activation. The mRNA and protein levels of KITL were significantly higher after 24 h of culture (Fig. 3e and Supplementary Fig. S3), and the levels of p-Akt and p-FOXO3a were significantly higher after 48 h of culture (Fig. 3d) in the pyruvate-free group than the control. Furthermore, the number of oocytes with FOXO3a nuclear export was significantly increased in the pyruvate-free group (14.72%) compared with that in the control group (8.79%; Fig. 3f, g). In contrast, the levels of p-Akt and p-FOXO3a and the number of oocytes with FOXO3a nuclear export were significantly decreased in the pyruvate-free + 2-DG group compared with the pyruvate-free group (Fig. 3f–i). The number of growing follicles was also decreased (Supplementary Fig. S4a, b). The blockade of the communication network between pre-granulosa cells and oocyte by KIT inhibitor ISCK03 significantly decreased pyruvate-free-promoted p-Akt and p-FOXO3a levels and the number of growing follicles, but had no effect on pyruvate-free-promoted mTOR activity (Fig. 3j–l). These results indicate that short-term pyruvate deprivation promotes mTOR and then KIT-Akt-FOXO3a signaling through enhanced glycolysis in mouse ovaries, resulting in primordial follicle activation.

### Short-term pyruvate deprivation increases the number of developing follicles in the mouse

**Fig. 4:**
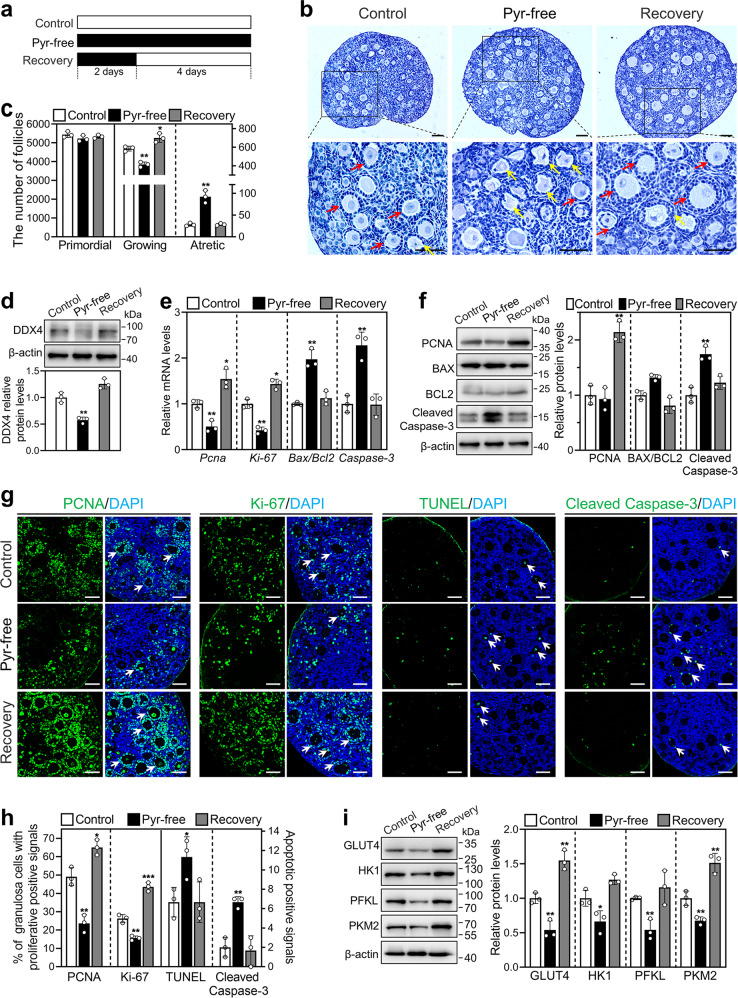
Effect of short-term pyruvate deprivation on the development of mouse follicles. **a** The timeline of the experiment was shown. Ovaries at 2 dpp were cultured in standard (control) or pyruvate-free (pyr-free) medium for 6 days, or cultured in pyruvate-free medium for 2 days and then in standard medium for 4 days (recovery). **b**, **c** Morphological comparison of the ovaries (**b**) and the number of primordial, growing (red arrows, **c**) and atretic follicles (yellow arrows, **c**) in the control, pyruvate-free and recovery groups. Nuclei were stained by hematoxylin. Scale bars: 50 μm. **d** DDX4 protein levels in the control, pyruvate-free, and recovery groups. **e** The mRNA levels of *Pcna*, *Ki-67*, *Bax/Bcl-2*, and *Caspase-3* in the control, pyruvate-free and recovery groups. **f** The protein levels of PCNA, BAX, BCL-2, and Cleaved Caspase-3 in the control, pyruvate-free and recovery groups. **g** Immunofluorescence stain of PCNA, Ki-67, TUNEL and Cleaved Caspase-3 (green) in the control, pyruvate-free and recovery groups. DAPI, blue. Arrows show the cells with positive signals. Scale bars: 50 μm. **h** The percentage of granulosa cells with PCNA- and Ki-67-positive signals, and the number of cells with TUNEL- and Cleaved Caspase-3-positive signals in each section in the control, pyruvate-free and recovery groups. **i** The protein levels of GLUT4, HK1, PFKL, and PKM2 in the control, pyruvate-free and recovery groups. All the experiments were repeated three times, and the representative images are shown. In western blot results, β-actin was used as an internal control. Bars indicate the mean ± SD. *P < 0.05, **P < 0.01, and ***P < 0.001 vs. control.

To explore the effect of short-term pyruvate deprivation on follicle development, we cultured neonatal mouse ovaries in standard medium (control) for 6 days, or in the pyruvate-free medium for 2 days and then in standard medium for 4 days (recovery group; Fig. 4a). The number of growing follicles was significantly increased in the recovery group (700.00) compared with the control group (585.00; Fig. 4b, c). The levels of DDX4 were also increased in the recovery group compared with the control group (Fig. 4d). There was no difference in the number of atretic follicles between these two groups (Fig. 4b, c).

To accurately judge the growth status of the follicles, we analyzed the expression and location of several proliferation and apoptosis markers. The levels of proliferation-related genes (*Pcna* and *Ki-67*) and protein (PCNA) and the percentage of granulosa cells with PCNA- and Ki-67-positive signals were significantly increased in the recovery group compared with those in the control group (PCNA: 65.04% vs. 49.10%; Ki-67: 43.59% vs. 26.28%; Fig. 4e–h). In addition, the levels of GLUT4 and PKM2 were significantly increased in the recovery group compared with those in the control group (Fig. 4i), consistent with follicle development. However, the levels of apoptosis-related genes and proteins and the number of cells with cleaved caspase-3- and TUNEL-positive signals were not different between the control and recovery groups (Fig. 4e–h). These results indicate that follicles activated by short-term pyruvate deprivation can continue to grow without atresia.

However, compared with that of the control, long-term pyruvate deprivation lasting up to 6 days (pyr-free group. Figure 4a) significantly decreased the number of growing follicles (Fig. 4b, c), the number of granulosa cells with proliferation-positive signals (Fig. 4g, h), and the levels of proliferation- and glycolysis-related genes and/or proteins (Fig. 4e, f, i). Consistent with this, long-term pyruvate deprivation significantly increased the number of atretic follicles (Fig. 4b, c), the number of cells with apoptosis-positive signals (Fig. 4g, h), and the levels of apoptosis- and glycolysis-related genes and/or proteins (Fig. 4e, f, i). Thus, long-term pyruvate deprivation may cause metabolic damage and even apoptosis of cells, resulting in a decrease in growing follicles.

### Acute fasting promotes glycolysis and primordial follicle activation in mouse ovaries

**Fig. 5:**
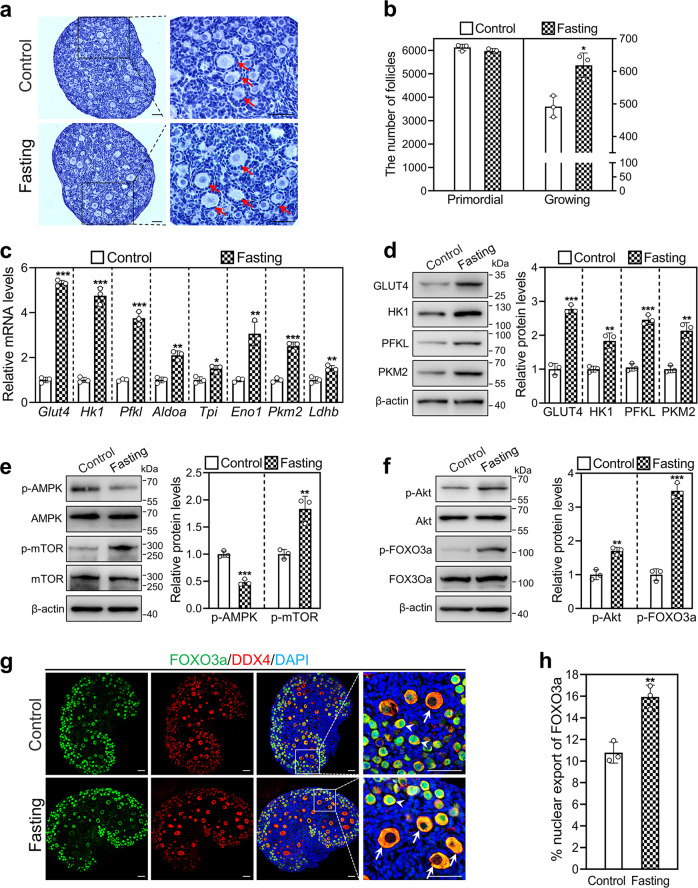
Effect of acute fasting on glycolysis and mouse primordial follicle activation in vivo. Two-day-old female mice were kept with their mother (control), or were separated from their mother for 18 h and then returned to their mother (acute fasting). The ovaries were collected at 24 h (**c**–**f**) and 48 h (**a**, **b**, **g**, **h**) of treatment, respectively. **a**, **b** Morphological comparison of the ovaries (**a**) and the number of primordial and growing follicles (arrows, **b**) in the control and acute fasting group. Nuclei were stained by hematoxylin. Scale bars: 50 μm. **c** The mRNA levels of *Glut4*, *Hk1*, *Pfkl*, *Aldoa*, *Eno1*, *Tpi*, *Pkm2*, and *Ldhb* in the control and acute fasting group. **d** The protein levels of GLUT4, HK1, PFKL, and PKM2 in the control and acute fasting group. **e**, **f** The protein levels of p-AMPK, p-mTOR (**e**), p-Akt, and p-FOXO3a (**f**) in the control and acute fasting group. **g**, **h** The localization of FOXO3a in oocyte nuclear (arrowheads) and cytoplasm (arrows, **g**) and the percentage of oocytes with FOXO3a nuclear export (**h**) in each section in the control and acute fasting group. FOXO3a, green; DDX4, red; DAPI, blue. Scale bars: 50 μm. Fasting, acute fasting. All the experiments were repeated three times, and the representative images are shown. In western blot results, the levels of total AMPK, mTOR, Akt, and FOXO3a were used as the corresponding internal control for p-AMPK, p-mTOR, p-Akt, and p-FOXO3a, respectively, and β-actin was used as the internal control for GLUT4, HK1, PFKL, and PKM2. Bars indicate the mean ± SD. *P < 0.05, **P < 0.01, and ***P < 0.001 vs. control.

Previous studies reported that refeeding after fasting increases glycolysis in rat liver [23]. We studied the effect of acute fasting on glycolysis and primordial follicle activation in vivo. The number of growing follicles in the acute fasting group (618.33) was significantly increased compared with that in the control group (491.67; Fig. 5a, b). The levels of mRNAs (*Glut4*, *Hk1*, *Pfkl*, *Aldoa*, *Eno1*, *Tpi*, *Pkm2*, and *Ldhb*) and proteins (GLUT4, HK1, PFKL, and PKM2) were significantly increased in the acute fasting group compared with the control group (Fig. 5c, d). Furthermore, p-AMPK was significantly decreased, and p-mTOR was significantly increased in the acute fasting group compared with the control group (Fig. 5e). Consistent with the activation of mTOR signaling, the levels of p-Akt and p-FOXO3a and the number of oocytes with FOXO3a nuclear export were significantly increased in the acute fasting group compared with the control group (Fig. 5f–h). These results suggest that enhanced glycolysis by acute fasting promotes mTOR signaling, resulting in mouse primordial follicle activation in vivo.

### Short-term pyruvate deprivation promotes the activation of human primordial follicles

**Fig. 6:**
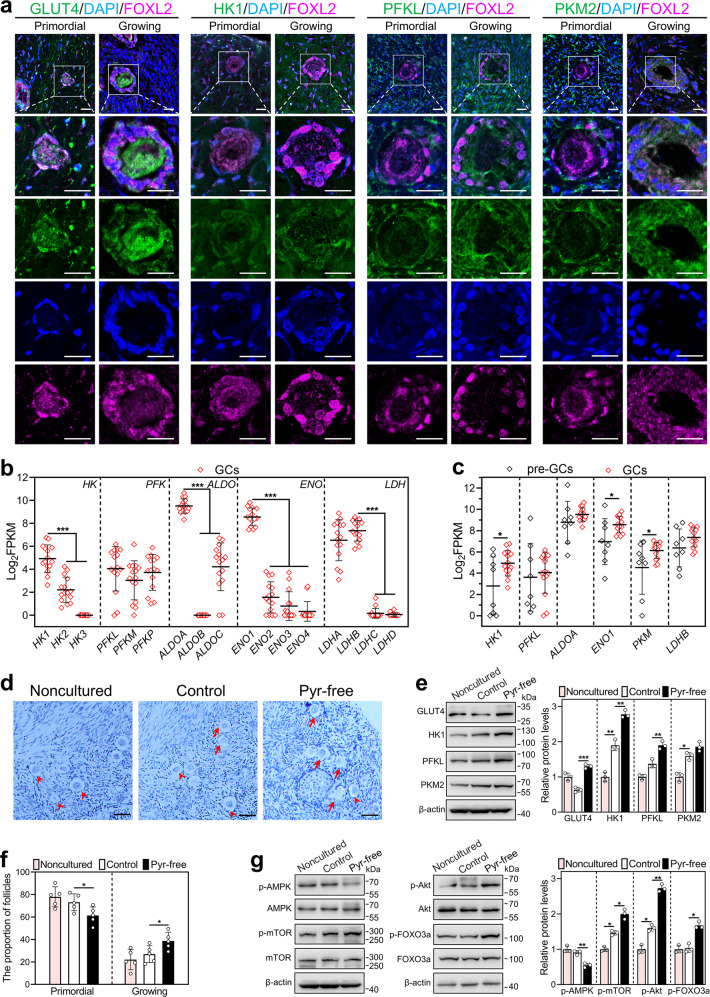
Effect of short-term pyruvate deprivation on human primordial follicle activation in vitro. **a** Immunofluorescence stain of GLUT4, HK1, PFKL, and PKM2 (green) in human primordial and growing follicles. FOXL2, purple; DAPI, blue. Scale bars: 20 μm. **b**, **c** Log_2_FPKM values were extracted from previously published data (GSE107746). The expression levels of isoforms in each family of *HK*, *PFK*, *ALDO*, *ENO,* and *LDH* in human granulosa cells of primary follicles (*n* = 15 follicles, **b**), and the expression levels of *HK1*, *PFKL*, *ALDOA*, *ENO1*, *PKM*, and *LDHB* in human granulosa cells of primordial (*n* = 8 follicles) and primary follicles (*n* = 15 follicles, **c**). GCs granulosa cells, pre-GCs pre-granulosa cells. **d**–**g** Human ovarian fragments were directly fixed in 4% PFA (noncultured), or cultured in standard medium (control), or cultured in the pyruvate-free medium for 2 days and then in standard medium for indicated days (pyruvate-free group). The fragments were collected after 3 days (**e**, **g**) and 6 days (**d**, **f**) of treatment, respectively. **d**, **f** Morphological comparison of human ovarian tissue fragments (**d**) and the proportion of primordial (arrowheads, **f**) and growing follicles (arrows, **f**) in the control and pyruvate-free group. (*n* = 5 independent experiments). Nuclei were stained by hematoxylin. Scale bars: 50 μm. **e** The protein levels of GLUT4, HK1, PFKL, and PKM2 in the noncultured, control, and pyruvate-free groups. **g** The protein levels of p-AMPK, p-mTOR, p-Akt, and p-FOXO3a in the noncultured, control, and pyruvate-free groups. The representative images are shown. In western blot results, the levels of total AMPK, mTOR, Akt, and FOXO3a were used as the corresponding internal control for p-AMPK, p-mTOR, p-Akt, and p-FOXO3a, respectively, and β-actin was used as the internal control for GLUT4, HK1, PFKL, and PKM2. Bars indicate the mean ± SD. *P < 0.05, **P < 0.01, and ***P < 0.001.

We further explored the expression characteristics of glycolysis-related proteins in human follicles. In the primordial follicles, GLUT4, HK1, PFKL, and PKM2 were localized in the pre-granulosa cells and oocytes (Fig. 6a). In the growing follicles, these proteins were localized in the granulosa cells, and only GLUT4 was also expressed in the oocytes (Fig. 6a). We analyzed the expression levels (assessment with FPKM) of glycolysis-related genes in human granulosa cells of primordial and primary follicles using published RNA-seq data (GEO: GSE107746) [24]. The mRNA levels of *HK1*, *PFKL*, *ALDOA*, *ENO1*, and *LDHB* in the granulosa cells of primary follicles were the highest (Fig. 6b), and the mRNA levels of *HK1*, *ENO1*, and *PKM* were significantly increased in the granulosa cells during the primordial-to-primary follicle transition (Fig. 6c). These results indicate that glycolysis is increased in human granulosa cells during the primordial-to-primary follicle transition.

Finally, we tested the effect of short-term pyruvate deprivation on human primordial follicle activation. The follicle counting showed that the proportion of growing follicles was significantly increased in the pyruvate-free group (38.70%) compared with that in the control group (26.90%; Fig. 6d, f). The protein levels of GLUT4, HK1, PFKL, and PKM2 were significantly increased in the pyruvate-free group compared with the control group (Fig. 6e). Furthermore, p-AMPK was significantly decreased, and p-mTOR, p-Akt, and p-FOXO3a were significantly increased in the pyruvate-free group compared with the control (Fig. 6g). These results suggest that short-term pyruvate deprivation promotes human primordial follicle activation by enhancing glycolysis and mTOR signaling. In addition, compared with the noncultured group, the levels of HK1, PKM2, p-mTOR, and p-Akt were significantly increased, and the number of growing follicles was slightly increased in the control group (Fig. 6d–g), suggesting the initiation of follicle growth in the cultured human fragments [25, 26].

## Discussion

The activation of primordial follicles is accompanied by an enhancement of energy metabolism. In this study, short-term pyruvate deprivation in the medium promoted mouse and human primordial follicle activation in vitro, and acute fasting facilitated mouse primordial follicle activation in vivo. The mechanism involved the activation of mTOR signaling by enhanced glycolysis in granulosa cells.

The activation of primordial follicles includes the proliferation of flattened granulosa cells and the growth of oocytes [6], and the energy needs are significant during the developmental transition [13]. In this study, glycolysis was significantly increased in neonatal mouse ovaries from 1 to 4 dpp, during which time primordial follicle activation is generally initiated [2]. GLUT4, HK1, PFKL, and PKM2 were expressed in mouse and human granulosa cells of primordial and growing follicles. However, HK1 and PFKL were not observed in mouse and human oocytes in the growing follicles, consistent with the view that the oocytes in growing follicles have no glycolytic capacity and depend on pyruvate secreted by granulosa cells [27]. In mice, these glycolysis-related proteins were significantly increased in granulosa cells during the primordial-to-primary follicle transition, suggesting that enhanced glycolysis in granulosa cells results in an increase in glycolysis in neonatal mouse ovaries. In humans, glucose degradation is enriched in the pre-granulosa cells of primordial follicles (Supplementary Fig. S5, the screenshot of transcriptome enrichment analysis in ref. [28]), and the mRNA levels of glycolysis-related genes are significantly increased in granulosa cells during primordial-to-primary follicle transition in our and other studies [28]. All of these results indicate that glycolysis is increased in granulosa cells but is decreased in oocytes during primordial follicle activation.

Interestingly, GLUT4, HK1, PFKL, and PKM2 were also expressed in mouse and human oocytes of primordial follicles. This is consistent with a previous study in which human oocytes of primordial follicles showed glycolytic capacity by RNA-seq analysis [29]. It is possible that a very small number of pre-granulosa cells in the primordial follicle have an inability to meet the metabolic requirements of the oocyte [13]. Only PKM2 and GLUT4 were expressed in mouse and human oocytes in all follicle stages, respectively. Their function in mouse or human oocytes needs further study. In addition, GLUT1 was weakly expressed in mouse preantral follicle cells, but was strongly expressed on the plasma membrane of antral follicle cells. This implies that GLUT1 may play an important role in the rapid development of antral follicles in mice.

Pyruvate is the final product of glycolysis, and cells in the pyruvate-free medium will experience a temporary energy shortage inside the cells. This energy shortage activates AMPK to increase catabolism, especially glycolysis [20], and enhanced glycolysis triggers the inhibition of AMPK and the subsequent activation of mTOR in the mouse brain [30]. In this study, the standard medium for culturing ovarian tissues contains pyruvate [10–12]. Short-term pyruvate deprivation promotes glycolysis via negative feedback of AMPK, and then enhanced glycolysis subsequently activates mTOR signaling by decreasing AMPK activity in mouse ovaries. Short-term pyruvate deprivation also promoted glycolysis and activated mTOR signaling by decreasing AMPK activity in human ovarian tissues. The energy sensor AMPK was obviously increased in mouse granulosa cells but decreased in oocytes (Supplementary Fig. S6), and enhanced glycolysis occurs in mouse and human granulosa cells during primordial follicle activation. Furthermore, the blockade of the communication network between pre-granulosa cells and oocyte by ISCK03 decreased pyruvate-free-promoted p-Akt and p-FOXO3a levels but had no effect on pyruvate-free-promoted mTOR activity in mouse ovaries. Thus, short-term pyruvate deprivation activates mTOR signaling by enhancing glycolysis in granulosa cells and then activates Akt-FOXO3a signaling in oocytes, resulting in mouse and human primordial follicle activation. In contrast, the blockade of glycolysis by 2-DG increased p-AMPK levels and decreased p-mTOR levels in mouse ovaries, resulting in the inhibition of primordial follicle activation, consistent with a previous study showing that glycolysis inhibitors increase p-AMPK levels and decrease p-mTOR levels in mouse glial cells [31].

In neonatal mice, refeeding after acute fasting increased glycolysis-related protein expression, decreased p-AMPK levels, and increased p-mTOR levels in the ovaries, consistent with previous studies showing that refeeding after starvation promotes glycolytic enzyme expression in rat liver [23] and increases mTOR signaling in mouse muscle [32, 33]. Enhanced glycolysis occurs in granulosa cells during primordial follicle activation. Thus, acute fasting activates mTOR signaling by enhanced glycolysis in granulosa cells, resulting in mouse primordial follicle activation in vivo.

Clinically, patients with metabolism disorders are related to both PCOS and POI [4, 34, 35]. PCOS is characterized by disordered follicle development including the accumulation of small preantral follicles, and patients often show a disorder of glycolysis and pyruvate metabolism in follicular fluids [36] and a heightened risk for diabetes [37]. The proportion of primordial follicles is decreased [5], and glycolysis is enhanced [38], but TCA (tricarboxylic acid cycle) is inhibited in PCOS patients [38, 39]. Thus, enhanced glycolysis may increase recruitment from the primordial follicle pool, resulting in a higher density of small preantral follicles in PCOS patients. POI is not only induced by genetic and iatrogenic factors but is also associated with autoimmune conditions, such as hypothyroidism [40, 41] and diabetes [42]. It has been reported that a downregulated lncRNA-ALDOA in granulosa cells and the gene variant of HK3 are associated with POI [43, 44]. Thus, a decrease in glycolytic capacity could result in POI via a decrease in the number of growing follicles.

**Fig. 7:**
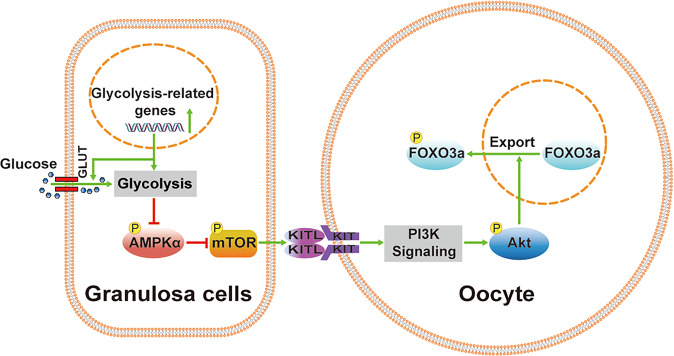
The proposed model of glycolysis in primordial follicle activation. Enhanced glycolysis in granulosa cells activates mTOR signaling by decreasing AMPK activity. This process triggers the transduction of KITL signaling in pre-granulosa cells to PI3K-Akt-FOXO3a signaling in oocytes, resulting in the activation of dormant primordial follicles.

In conclusion, enhanced glycolysis in granulosa cells by short-term pyruvate deprivation or acute fasting activated mTOR signaling and downstream pathways, resulting in mouse and human primordial follicle activation (Fig. 7). Thus, glycolytic activity in granulosa cells is important for the recruitment of primordial follicles and the further development of growing follicles. Glycolytic disorders are associated with PCOS and POI. Our findings not only contribute to a better understanding of the role of glycolysis in primordial follicle activation but also have potential implications for the diagnosis and treatment of clinical infertility.

## Materials and methods

### Animals and chemicals

ICR (CD1) mice at the age of 8–12 weeks were purchased from the Guangdong Medical Laboratory Animal Center (Guangzhou, China), and housed in individually ventilated cages at 22 ± 1 °C and 50–70% humidity. Animals were kept in a 12 h light–dark cycle and fed freely available water and food unless stated otherwise. Adult female and male mice were paired one-to-one to generate neonatal mice. The day after partum was designated as 0.5 days postpartum (dpp). For the acute fasting protocols, 2-day-old female mice with the same bodyweight (2.5 ± 0.1 g) were randomly divided into two groups: (1) mice in the control group were kept with their mother (ad libitum access to milk) all the time; (2) mice in the acute fasting group were separated from their mother for 18 h and then returned to their mother. All animal procedures were conducted in accordance with the Guidelines of the Animal Care and Use Committee of South China University of Technology. Unless otherwise stated, the reagents were purchased from Sigma-Aldrich (St. Louis, MO, USA).

### Mouse ovary culture

The ovaries were isolated from newborn mice at the designated time by a pair of 26-gauge needles in phosphate-buffered saline (PBS) under a stereomicroscope (Carl Zeiss, stemi 508, Göttingen, Germany). The ovaries were cultured on a Millipore insert (PICMORG50, Millipore, Billerica, MA, USA) in a six-well culture plate (NEST Biotechnology, Beijing, China) containing the indicated culture medium. The standard medium for ovary tissue culture was Dulbecco’s modified Eagle’s medium (DMEM, containing 1 mM pyruvate) with nutrient mixture F-12 (DMEM/F-12. Thermo Fisher Scientific, Waltham, MA, USA), supplemented with 100 UI/ml penicillin–streptomycin, 1% insulin–transferrin–selenium (ITS) and 3 mg/ml bovine serum albumin (BSA). In pyruvate-free medium, DMEM without pyruvate was used. In some experiments, the ovaries were cultured in pyruvate-free medium, supplemented with 10 mM 2-DG or 5 μM ISCK03. The ovaries were incubated at 37 °C with 5% CO_2_ and saturated humidity, and the medium was changed every 2 days.

### Human ovarian tissue culture

Six women aged 31–44 years (36.7 ± 5 years) donated small ovarian cortical biopsy specimens (adjacent non-tumor tissue) while undergoing routine gynecological laparoscopies at Shunde Hospital, Southern Medical University (The First People’s Hospital of Shunde), Foshan, Guangdong, China. Written informed consents were obtained before surgery. This study was performed in accordance with the Declaration of Helsinki. Approval for this study was obtained from the Ethical Committee of Shunde Hospital, Southern Medical University (No. 20210703). Human ovarian tissues were placed in pre-equilibrated Dulbecco’s phosphate-buffered saline (DPBS) supplemented with penicillin–streptomycin and ITS and were immediately transported to the laboratory. Under sterile conditions, each ovarian tissue was divided into fragments of ~1 mm^3^. Several fragments from each tissue were fixed in 4% paraformaldehyde (PFA, Santa Cruz, Texas, USA) and stained with hematoxylin to evaluate the follicle density (noncultured control at Day 0). The remaining fragments were randomly divided equally, and cultured in standard medium (control), or cultured in pyruvate-free medium for 2 days and then in standard medium for indicated days (pyruvate-free group). The fragments were placed on a Millipore insert in a six-well culture plate and incubated at 37 °C with 5% CO_2_ and saturated humidity, and the medium was changed every 2 days. The human cortex fragments were collected to analyze the protein levels after 3 days of culture or to count the follicle number after 6 days of culture.

### Histological and morphological analysis

The ovarian tissues were fixed in 4% PFA for 12 h, dehydrated with an alcohol gradient, embedded in paraffin (Leica Biosystems, Wetzlar, Germany), serially sectioned at a thickness of 5 μm and mounted on glass slides. After drying at 42 °C for 12 h, the sections were deparaffinized in xylene, hydrated by gradient alcohol, and stained with hematoxylin (Solarbio, Beijing, China). The follicles with a clearly visible nucleus were counted. These follicles included primordial follicles (an oocyte surrounded by a few flat pre-granulosa cells), growing follicles (an enlarged oocyte surrounded by more than one cuboidal granulosa cell), and atretic follicles (a degenerating oocyte with shrunken cytoplasm).

In mouse ovaries, the primordial follicles were counted in every fifth section and multiplied by a correction factor of 5 to calculate the number of all primordial follicles in each ovary. The growing and atretic follicles were counted by examining serial sections of the entire ovaries. In human ovarian tissues, primordial and growing follicles were counted by examining serial sections of entire ovarian fragments. Follicle density was defined as the number of follicles per mm^3^ and a mean value was obtained from all sections. The follicle density = the total number of follicles/tissue volume. And, tissue volume was calculated as the sum of the area of all sections multiplied by the distance between sections (0.005 mm) [25]. All the follicle counting were completed by the blinded observers.

### Immunofluorescent staining

The ovarian tissues were embedded in paraffin and cut into 5-μm sections as described above. After drying for 12 h at 42 °C, the sections were deparaffinized, hydrated, and subjected to antigen retrieval with sodium citrate buffer in a microwave at 95 °C. The sections were blocked with 10% donkey serum for 1 h at room temperature, incubated with primary antibodies (Supplementary Table S1) for 12 h at 4 °C, and then incubated with Alexa Fluor 488- or 555-conjugated secondary antibodies (Thermo Fisher Scientific) for 1 h at 37 °C. Finally, the sections were stained with 4′,6-diamidino2-phenylindole (DAPI) for 5 min and mounted on slides with anti-fluorescence quenching agents (Ruitaibio, Beijing, China). Sections were photographed using a Zeiss LSM 800 confocal microscope (Carl Zeiss, Oberkochen, Germany), and the fluorescence intensity was analyzed using Zeiss Zen 3.0 software (Carl Zeiss). The relative fluorescence intensity was calculated by dividing the fluorescence intensity of cells by the fluorescence intensity of the background. Here we used the fluorescence intensity of the oocyte nucleus as the background.

In addition, the five central sections in each ovary were used to analyze the percentage of primordial follicles or granulosa cells with positive signals, and the percentage of oocytes with FOXO3a nuclear export. The mean percentage in five sections was calculated as the percentage of follicles/cells in one section for one independent sample. Primordial follicles containing more than one granulosa cells with proliferation-positive signal were considered to be proliferation-positive primordial follicles. All the positive signal and FOXO3a counts were completed by the blinded observers.

### Western blotting analysis

Total protein from six mouse ovaries of each treatment was extracted using WIP buffer (Cell Chip Biotechnology, Beijing, China) with 1 mM phenylmethylsulfonyl fluoride (PMSF, Cell Signaling Technologies, Boston, MA, USA), and the protein concentration was detected by bicinchoninic acid (BCA) assay (Beyotime, Beijing, China). Equivalent amounts of protein (30 μg) containing sodium dodecyl sulfate (SDS) buffer were separated in a 10% SDS-polyacrylamide gel by electrophoresis and then transferred to pure polyvinylidene fluoride (PVDF) membranes (Millipore). The membranes were placed in 5% nonfat milk for 1 h to block nonspecific binding and then incubated with the corresponding primary antibodies (Supplementary Table S1) for 12 h at 4 °C. After rinsing with TBST, the membranes were then incubated with anti-rabbit or anti-mouse IgG secondary antibody (diluted 1:5000, ZSGB-BIO, Beijing, China). Finally, the membranes were visualized using SuperSignal West Pico Chemiluminescent Substrate (Thermo Fisher Scientific) and imaged by the Tanon 5200 chemiluminescent imaging system (Tanon, Shanghai, China). The band density was quantified using ImageJ software (NIH Image, Bethesda, MD, USA). The β-actin was used as an internal control. Uncropped scans of the most important blots are shown in Supplementary Fig. S7.

### RNA extraction and qRT-PCR

Total RNA from six mouse ovaries of each treatment was extracted using the ReliaPrep™ RNA Tissue Miniprep System (Promega, Madison, WI, USA). The quality and quantity of the RNA were determined using a NanoDrop™ One Spectrophotometer (Thermo Fisher Scientific). Then, the cDNA was synthesized from 200 μg of RNA using the GoScript™ Reverse Transcription System (Promega) according to the manufacturer’s guidelines. qRT-PCR was conducted using a Light Cycler 96 instrument (Roche, Basel, Switzerland). Ribosomal protein L19 (*Rpl19*) was used to normalize the data, and gene expression levels were calculated using the comparative cycle threshold (2^−△△Ct^) method. The primers were obtained from BGI Genomics (BGI-Tech, Shenzhen, China), and the sequences are listed in Supplementary Table S2.

### BrdU and TUNEL assay

In the bromodeoxyuridine (BrdU) incorporation assay, the mouse ovaries were incubated in a culture system supplemented with 10 μM BrdU for 2 h, collected, fixed, and finally cut into 5 μm paraffin sections. The five central sections in each ovary were incubated with anti-BrdU antibody for 12 h and then with Alexa Fluor 488-conjugated donkey secondary antibody for 2 h. The detailed steps of deparaffinization, antigen retrieval, serum blocking, and final DAPI counterstaining were carried out as described above.

In the terminal deoxynucleotidyl transferase-mediated deoxyuridine triphosphate (TUNEL) assay, the ovarian sections from each treatment were treated using the Click-iT Plus TUNEL Assay (Thermo Fisher Scientific). In short, deparaffinized sections were fixed in 4% PFA for 15 min, and permeabilized in proteinase K solution for 30 min. Then, the sections were incorporated with EdUTP by incubating with the TdT reaction mixture for 60 min and labeled with Alexa Fluor 488 dyes by covering the Click-iT™ Plus TUNEL reaction cocktail in the dark for 60 min. After adding 3% BSA in PBS for 5 min, the sections were counterstained with DAPI for 2 min. Finally, the images were captured by a Zeiss LSM 800 confocal microscope (Carl Zeiss).

### Statistical analysis

All experiments were carried out at least three times to obtain valid data. The data were analyzed and graphed using GraphPad Prism software (v8.3.0, La Jolla, CA, USA). The statistical significance between two groups was analyzed by two-tailed unpaired Student’s t-tests (*P < 0.05, **P < 0.01, ***P < 0.001).

## Supplementary information

Supplementary Figure Legends and Tables

checklist

Figure S1

Figure S2

Figure S3

Figure S4

Figure S5

Figure S6

Figure S7

## Data Availability

All data generated or analyzed during this study are available from the corresponding author on reasonable request.
