# Supplementary material for: Enhanced glycolysis in granulosa cells promotes the activation of primordial follicles through mTOR signaling
Source: Cell Death Dis. 2022 Jan 27;13(1):87. doi: 10.1038/s41419-022-04541-1 (PMC8795455; doi:10.1038/s41419-022-04541-1)
Supplement: Supplementary file 1 — Supplementary Figure Legends and Tables [file 41419_2022_4541_MOESM1_ESM.docx]

**Supplemental Material**

Xiaodan Zhang, et al.

**Enhanced glycolysis in granulosa cells promotes the activation of primordial follicles through mTOR signaling**

**Supplementary Figure Legends**

**Fig. S1. Localization of GLUT1 in neonatal mouse ovaries.** Immunofluorescence stain of GLUT1 in the ovaries at 1, 4 and 7 dpp. GLUT1, green; DDX4, red; DAPI, blue. (n = 3 independent experiments, and the representative images are shown). Scale bars: 50 μm.

**Fig. S2. Localization of glycolysis-related proteins in adult mouse ovaries.** Immunofluorescence stain of GLUT1, GLUT4, HK1, PFKL and PKM2 in the ovaries from adult mice (2-3 months). Glycolysis-related proteins, green; DDX4, red; DAPI, blue. (n = 3 independent experiments, and the representative images are shown). Scale bars: 50 μm.

**Fig. S3. Quantitative RT-PCR analysis of glycolysis-related genes and *Kitl* in cultured mouse ovaries.** Ovaries at 2 dpp were cultured in standard (control) or pyruvate-free (pyr-free) medium for 24 h. Comparison of the mRNA levels of *Glut4*, *Hk1*, *Pfkl*, *Aldoa*, *Eno1*, *Tpi*, *Pkm2*, *Ldhb* and *Kitl* in the control and pyruvate-free group. (n = 3 independent experiments). Bars indicate the mean ± SD. **P < 0.01 and ***P < 0.001 vs. control.

**Fig. S4. Effect of 2-DG on the activation of mouse primordial follicles.** Ovaries at 2 dpp were cultured in standard (control), pyruvate-free (pyr-free) or pyruvate-free supplemented with 10 mM 2-DG (pyr-free + 2-DG) medium for 48 h. Morphological comparison of the ovaries (**a**) and the number of primordial and growing follicles (arrows. **b**) in the control, pyruvate-free and pyruvate-free + 2-DG groups. Nuclei were stained by hematoxylin. 2-DG, 2-deoxyglucose. Scale bars: 50 μm. All the experiments were repeated three times, and the representative images are shown. Bars indicate the mean ± SD. *P <0.05, **P < 0.01 and ***P < 0.001.

**Fig. S5. Screenshot of Table II in ref. 28.** Glucose degradation is enriched in the pre-granulosa cells of human primordial follicles.

**Fig. S6. Localization of AMPK in neonatal mouse ovaries.** Immunofluorescence stain of AMPK in the ovaries at 1, 4 and 7 dpp. AMPK, green; DDX4, red; DAPI, blue. (n = 3 independent experiments, and the representative images are shown). The arrowheads and the arrows show the primordial and primary follicles, respectively. Scale bars: 50 μm.

**Fig. S7. Uncropped scans of the most important western blotting results**. **a** DDX4, BAX, BCL2 in the black dashed line box were used in Fig. 2d (DDX4) and in Fig. 2f (BAX and BCL2). **b** GLUT4, HK1, PFKL and PKM2 in the black dashed line box were used in Fig. 3c. **c** p-TSC2, p-rpS6, p-S6K, KITL and p-FOXO3a in the black dashed line box were used in Fig. 3e (p-TSC2, p-rpS6, p-S6K and KITL) and in Fig. 3d (p-FOXO3a). **d** p-AMPK, p-Akt in the black dashed line box was used Fig. 5e (p-AMPK) and in Fig. 5f (p-Akt). **e** PCNA in the black dashed line box was used in Fig. 2f. **f** Cleaved Caspase-3 in the black dashed line box was used in Fig. 4f. **g** p-mTOR in the black dashed line box was used in Fig. 5e.

**Supplementary Tables**

**Table S1. List of primary antibodies used in immune detection.**

| Antibody | Catalog Code | Source | Host | Dilution | |
| --- | --- | --- | --- | --- | --- |
|  |  |  |  | IF | WB |
| Akt | 4691 | Cell Signaling Technology | Rabbit |  | 1:1000 |
| p-Akt | 4060 | Cell Signaling Technology | Rabbit |  | 1:1000 |
| AMPK | ab131512 | Abcam | Rabbit | 1:200 | 1:1000 |
| p-AMPK | 2535 | Cell Signaling Technology | Rabbit |  | 1:1000 |
| BrdU | ab1893 | Abcam | Sheep | 1:200 |  |
| BAX | 50599-2-Ig | Proteintech | Rabbit |  | 1:1000 |
| BCL2 | 26593-1-AP | Proteintech | Rabbit |  | 1:1000 |
| Cleaved Caspase-3 | 9664 | Cell Signaling Technology | Rabbit | 1:50 | 1:1000 |
| DDX4 | ab27591 | Abcam | Mouse | 1:200 | 1:1000 |
| FOXL2 | NB100-1277 | Novus Biologicals | Goat | 1:300 |  |
| FOXO3a | 12829 | Cell Signaling Technology |  | 1:100 | 1:1000 |
| p-FOXO3a | ab26649 | Abcam | Rabbit |  | 1:1000 |
| GLUT1 | ab115730 | Abcam | Rabbit | 1:100 |  |
| GLUT4 | ab33780 | Abcam | Rabbit | 1:100 | 1:1000 |
| HK1 | ab150423 | Abcam | Rabbit | 1:100 | 1:1000 |
| Ki-67 | 9129s | Cell Signaling Technology | Rabbit | 1:100 |  |
| KITL | ab64677 | Abcam | Rabbit |  | 1:1000 |
| mTOR | 2972 | Cell Signaling Technology | Rabbit |  | 1:1000 |
| p-mTOR | 2971 | Cell Signaling Technology | Rabbit |  | 1:1000 |
| PCNA | 2586 | Cell Signaling Technology | Mouse | 1:100 | 1:1000 |
| PFKL | ab181064 | Abcam | Rabbit | 1:100 | 1:1000 |
| PKM2 | 4053 | Cell Signaling Technology | Rabbit | 1:100 | 1:1000 |
| RPS6 | ab40820 | Abcam | Rabbit |  | 1:1000 |
| p-RPS6 | ab215214 | Abcam | Rabbit |  | 1:1000 |
| S6K1 | ab9366 | Abcam | Rabbit |  | 1:1000 |
| P-S6K1 | ab228513 | Abcam | Rabbit |  | 1:1000 |
| TSC2 | 3612 | Cell Signaling Technology | Rabbit |  | 1:1000 |
| p-TSC2 | 3611 | Cell Signaling Technology | Rabbit |  | 1:1000 |
| β-actin | 4967 | Cell Signaling Technology | Rabbit |  | 1:1000 |

IF: Immunofluorescence; WB: Western blotting

**Table S2. Primers for qRT-PCR**

| Genes | Forwards (5’-3’) | Backwards (5’-3’) |
| --- | --- | --- |
| *Aldoa* | CGTGTGAATCCCTGCATTGG | CAGCCCCTGGGTAGTTGTC |
| *Aldob* | GAAACCGCCTGCAAAGGATAA | GAGGGTCTCGTGGAAAAGGAT |
| *Aldoc* | AGAAGGAGTTGTCGGATATTGCT | TTCTCCACCCCAATTTGGCTC |
| *Bax* | TTTCATCCAGGATCGAGCAGG | GCAAAGTAGAAGAGGGCAACCAC |
| *Bcl2* | CTACCGTCGTGACTTCGCA | TACCCAGCCTCCGTTATCC |
| *Caspase-3* | CCGGTTACTATTCCTGGAGA | TAACACGAGTGAGGATGTGC |
| *Eno1* | TGCGTCCACTGGCATCTAC | CAGAGCAGGCGCAATAGTTTTA |
| *Eno2* | AGGTGGATCTCTATACTGCCAAA | GTCCCCATCCCTTAGTTCCAG |
| *Eno3* | CACAGCCAAGGGTCGATTCC | CCCAGGTATCGTGCTTTGTCT |
| *Eno4* | CAGGCGATGGCGTACTACC | CAGGTGCCCGTAGACATCC |
| *Gdf9* | TCTTAGTAGCCTTAGCTCTCAGG | TGTCAGTCCCATCTACAGGCA |
| *Glut1* | CAGTTCGGCTATAACACTGGTG | GCCCCCGACAGAGAAGATG |
| *Glut2* | TCAGAAGACAAGATCACCGGA | GCTGGTGTGACTGTAAGTGGG |
| *Glut3* | ATGGGGACAACGAAGGTGAC | GTCTCAGGTGCATTGATGACTC |
| *Glut4* | ACACTGGTCCTAGCTGTATTCT | CCAGCCACGTTGCATTGTA |
| *Hk1* | CGGAATGGGGAGCCTTTGG | GCCTTCCTTATCCGTTTCAATGG |
| *Hk2* | TGATCGCCTGCTTATTCACGG | AACCGCCTAGAAATCTCCAGA |
| *Hk3* | TGCTGCCCACATACGTGAG | GCCTGTCAGTGTTACCCACAA |
| *Ki-67* | ATCATTGACCGCTCCTTTAGGT | GCTCGCCTTGATGGTTCCT |
| *Kitl* | GAATCTCCGAAGAGGCCAGAA | GCTGCAACAGGGGGTAACAT |
| *Ldha* | TGTCTCCAGCAAAGACTACTGT | GACTGTACTTGACAATGTTGGGA |
| *Ldhb* | CATTGCGTCCGTTGCAGATG | GGAGGAACAAGCTCCCGTG |
| *Ldhc* | CTGGTGCAAGAATGGTGTCTG | GGACAATGCCCGGAACAATG |
| *Ldhd* | CTGAAGGCAGTTGTAGGGAGC | GGAACACCTTGATTGTAGCACAG |
| *Pcna* | CGGCGTGAACCTGCAGAGCA | GGTTGCGGTCGCAGCGGTAT |
| *Pfkm* | TGTGGTCCGAGTTGGTATCTT | GCACTTCCAATCACTGTGCC |
| *Pfkl* | GAACTACGCACACTTGACCAT | CTCCAAAACAAAGGTCCTCTGG |
| *Pfkp* | GAAACATGAGGCGTTCTGTGT | CCCGGCACATTGTTGGAGA |
| *Pkm2* | GCCGCCTGGACATTGACTC | CCATGAGAGAAATTCAGCCGAG |
| *Rpl19* | CTGAAGGTCAAAGGGAATGTGTTC | TGGTCAGCCAGGAGCTTCTTG |
| *Tpi* | CCAGGAAGTTCTTCGTTGGGG | CAAAGTCGATGTAAGCGGTGG |
| *Zp3* | CCTCAGGACTAACCGTGTGGA | CCATCAGGCGAAGAGAGAAAG |
