# Supplementary figures and images for: Enhanced glycolysis in granulosa cells promotes the activation of primordial follicles through mTOR signaling
Source: Cell Death Dis. 2022 Jan 27;13(1):87. doi: 10.1038/s41419-022-04541-1 (PMC8795455; doi:10.1038/s41419-022-04541-1)

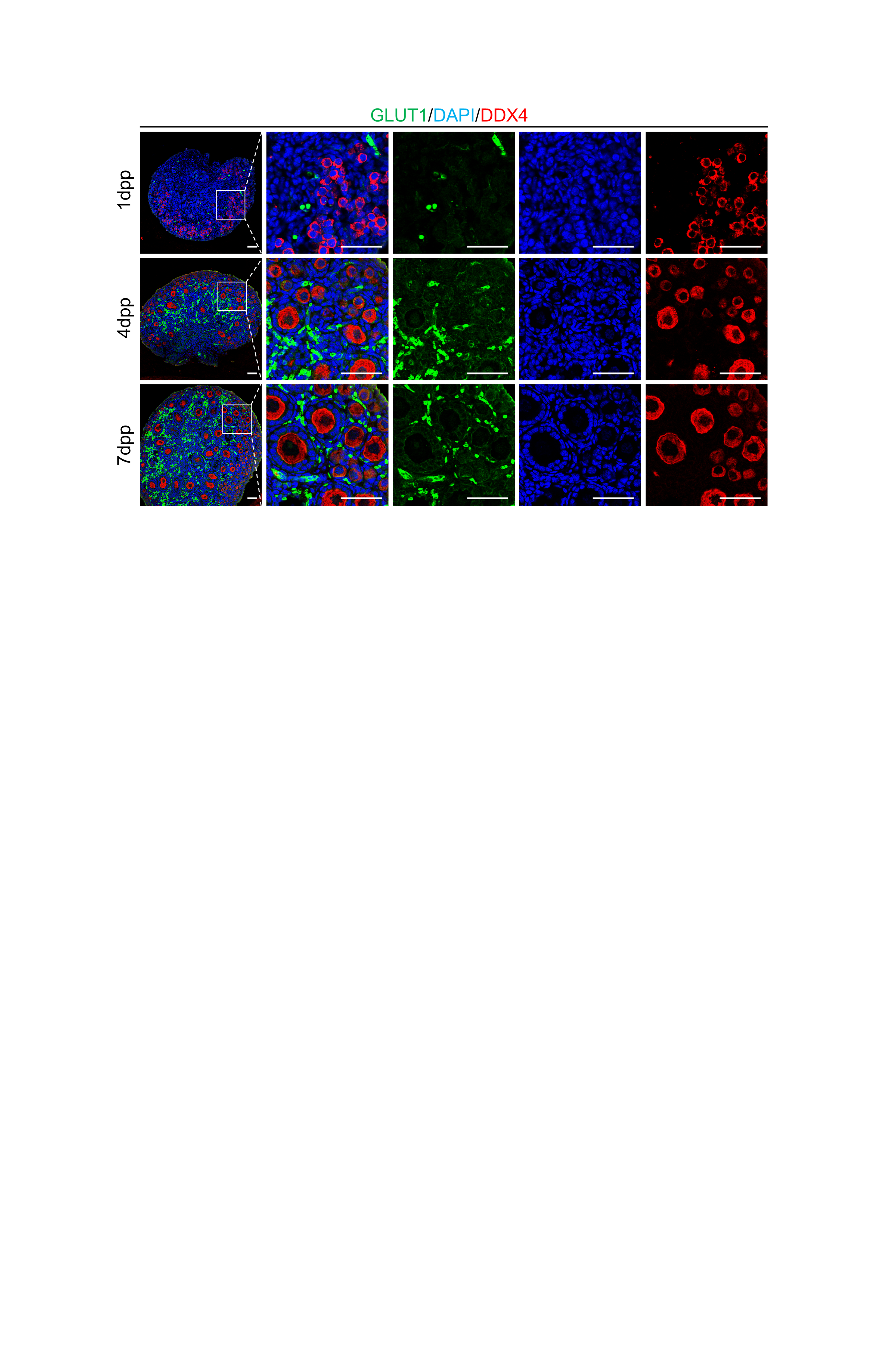

Supplement: Supplementary file 3 — Figure S1 [file 41419_2022_4541_MOESM3_ESM.tif]

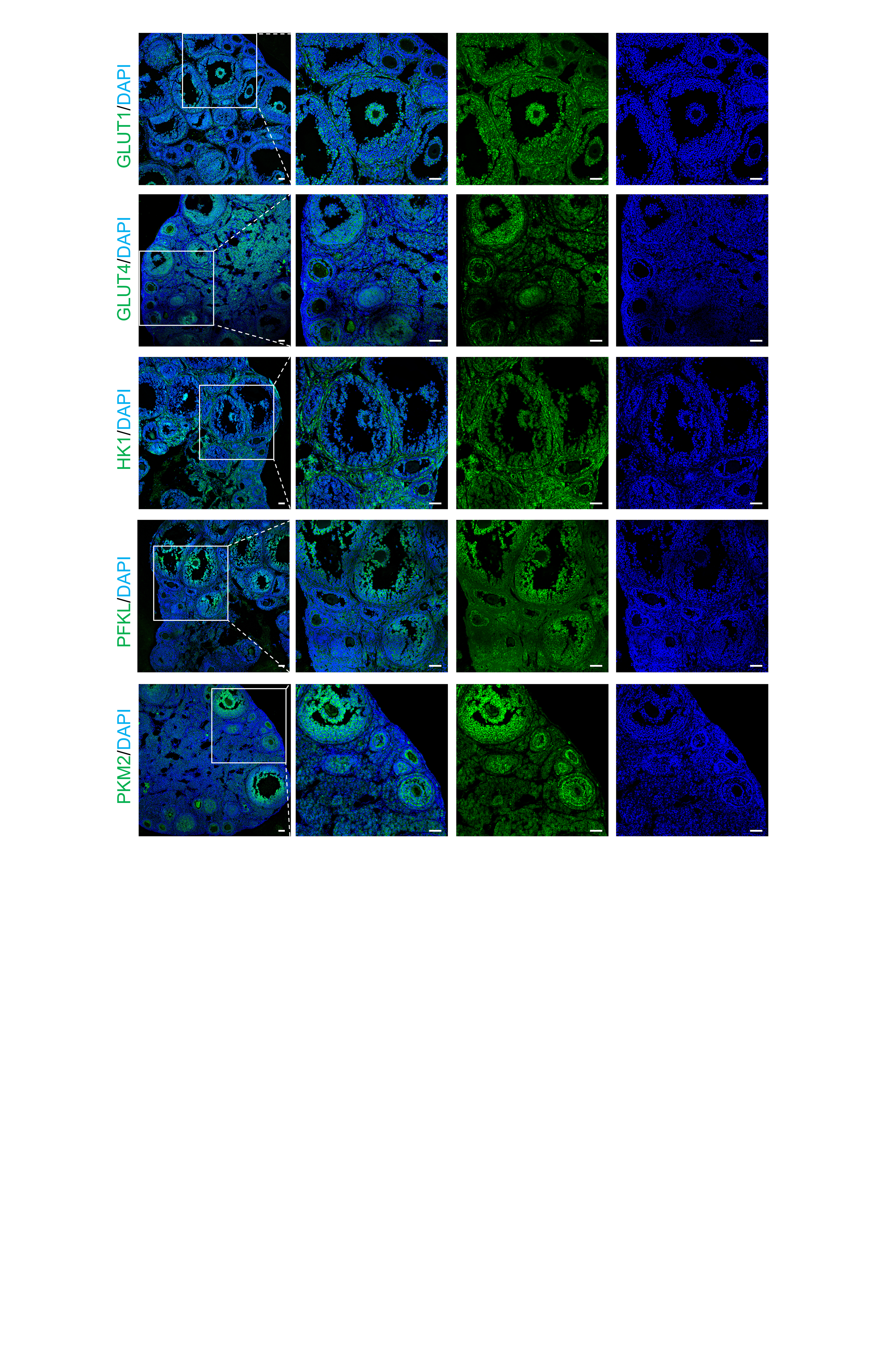

Supplement: Supplementary file 4 — Figure S2 [file 41419_2022_4541_MOESM4_ESM.tif]

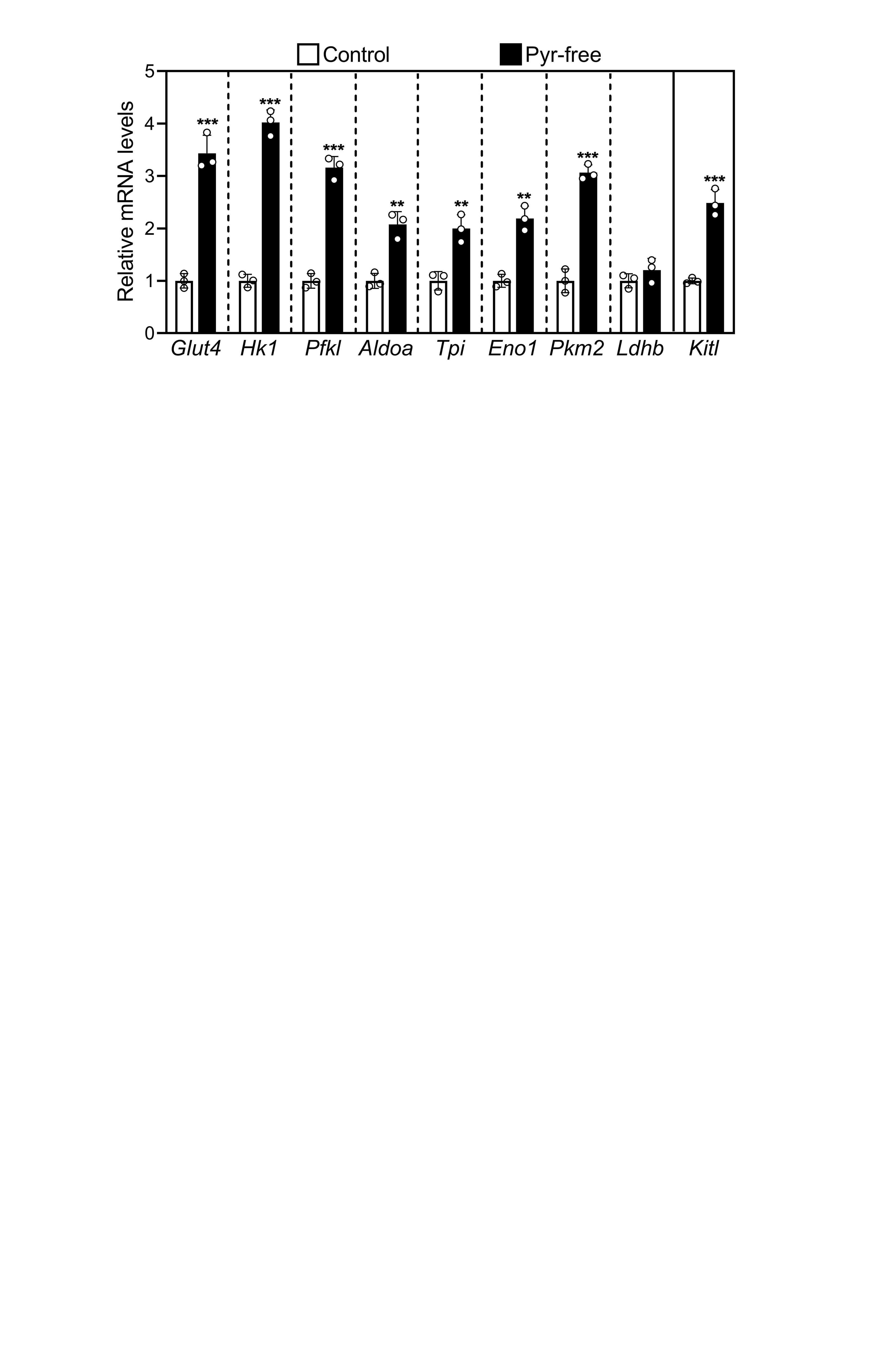

Supplement: Supplementary file 5 — Figure S3 [file 41419_2022_4541_MOESM5_ESM.tif]

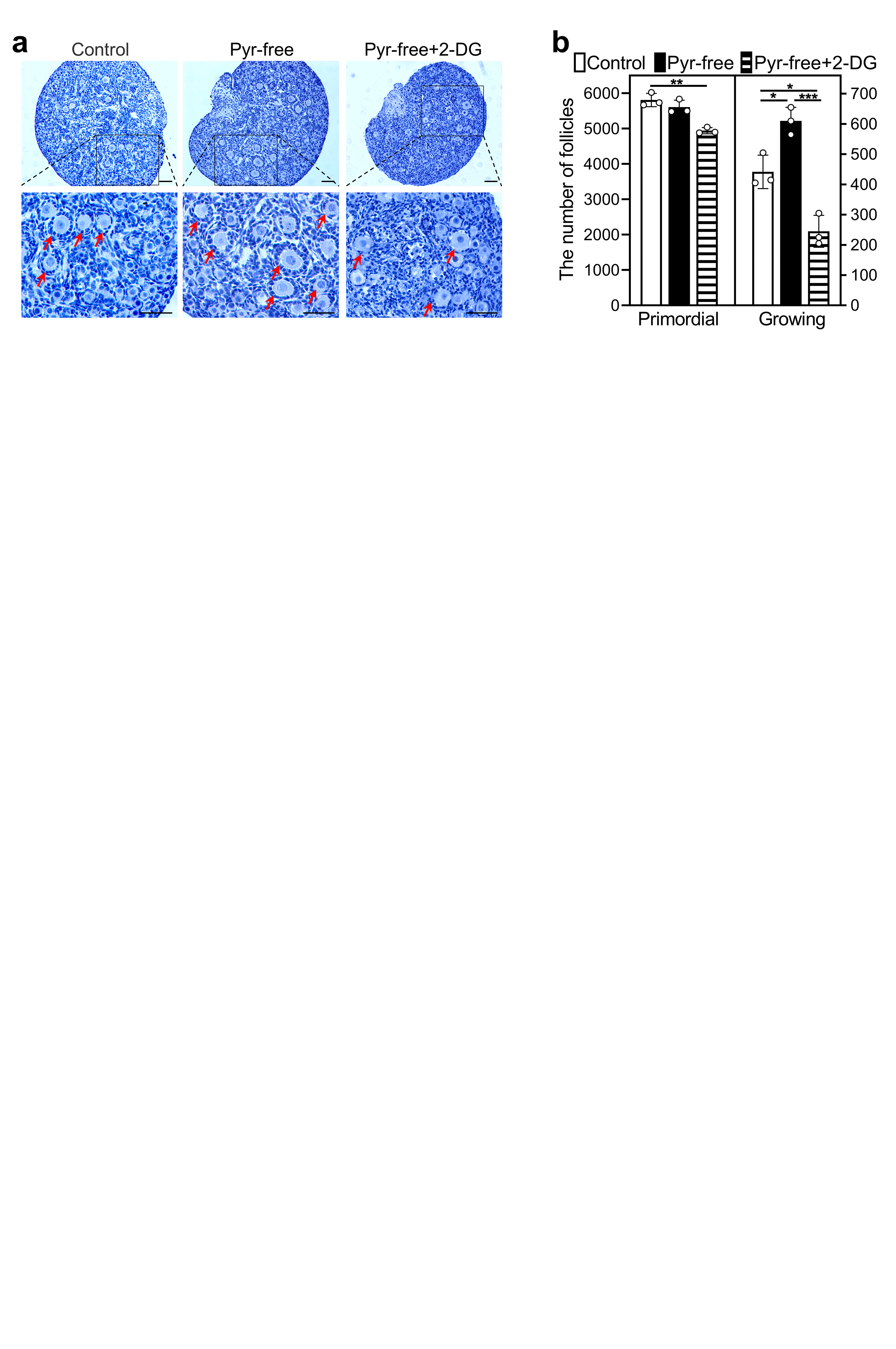

Supplement: Supplementary file 6 — Figure S4 [file 41419_2022_4541_MOESM6_ESM.tif]

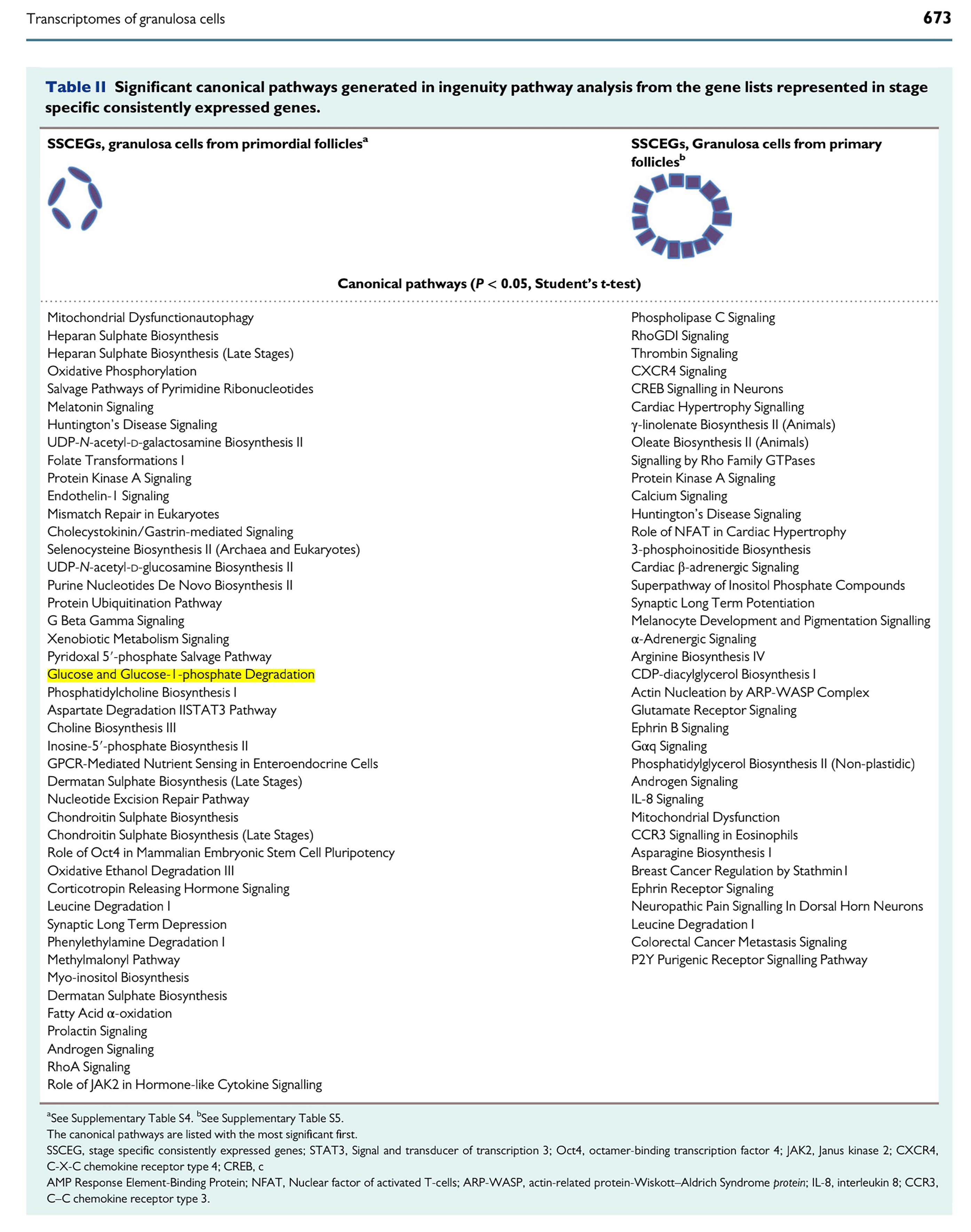

Supplement: Supplementary file 7 — Figure S5 [file 41419_2022_4541_MOESM7_ESM.tif]

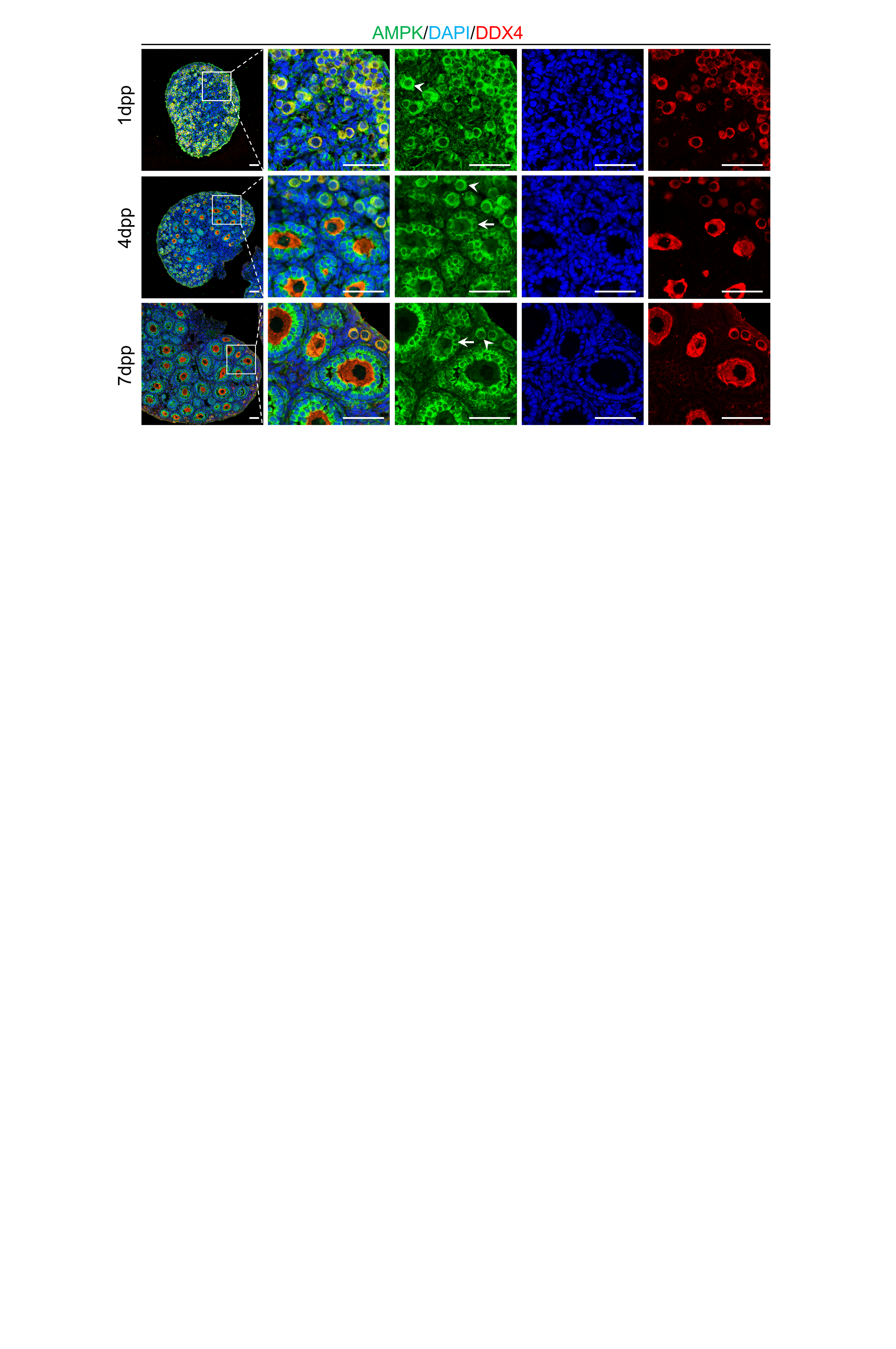

Supplement: Supplementary file 8 — Figure S6 [file 41419_2022_4541_MOESM8_ESM.tif]

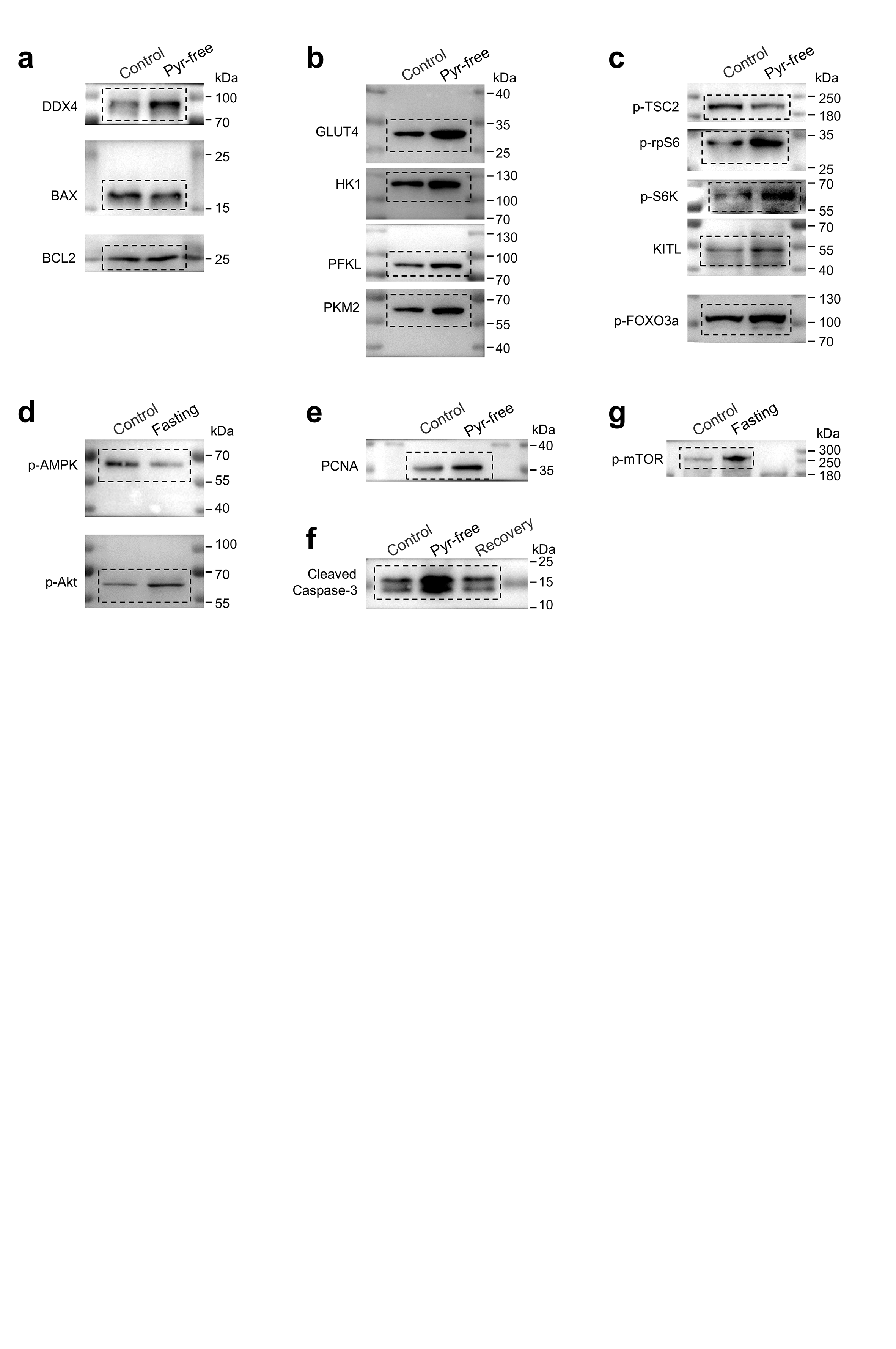

Supplement: Supplementary file 9 — Figure S7 [file 41419_2022_4541_MOESM9_ESM.tif]
